# Supplementary material for: Differences in neutralization susceptibility between clade C HIV viruses from breastmilk versus contemporaneous circulating viruses from sexually acquired infections
Source: bioRxiv. 2025 Nov 4:2025.11.04.686466. Preprint. [Version 1] doi: 10.1101/2025.11.04.686466 (PMC12637649; doi:10.1101/2025.11.04.686466)
Supplement: 1 [file NIHPP2025.11.04.686466v1-supplement-1.pdf]

bioRxiv preprint doi: https://doi.org/10.1101/2025.11.04.686466; this version posted November 4, 2025. The copyright holder for this preprint (which was not certified by peer review) is the author/funder, who has granted bioRxiv a license to display the preprint in perpetuity. It is made available under aCC-BY 4.0 International license.

Supplemental Figures.

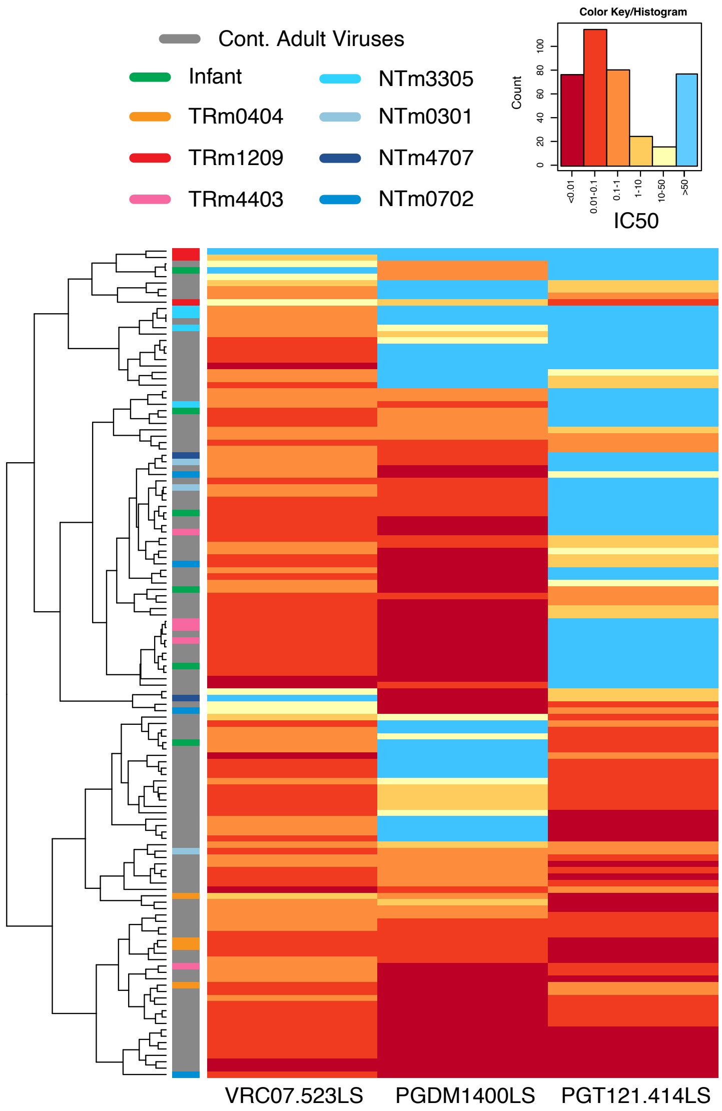

Fig S1. Heatmap visualization of the IC50 titers of all 130 Envs (rows) against the three bnAbs VRC07.523, PGT121, and PGDM1400 (columns). Titers are color-coded with warmer colors indicating stronger susceptibility and lighter colors indicating resistance, with light blue indicating above titers above the detection threshold of 50. Rows are hierarchically clustered and color-coded bars follow the same schema as in Fig. 1: warm colors for transmitting mothers, cold colors for non-transmitting mothers, and gray for contemporaneous adult viruses.

bioRxiv preprint doi: https://doi.org/10.1101/2025.11.04.686466; this version posted November 4, 2025. The copyright holder for this preprint (which was not certified by peer review) is the author/funder, who has granted bioRxiv a license to display the preprint in perpetuity. It is made available under aCC-BY 4.0 International license.

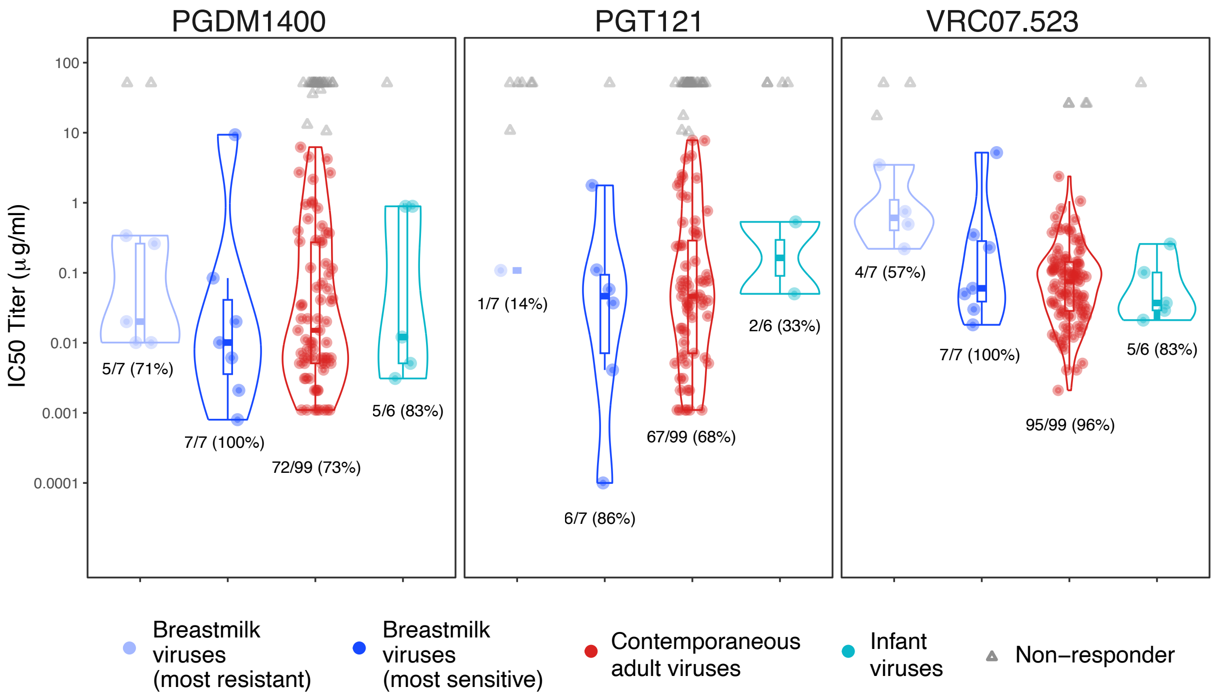

Fig S2. Distribution of IC50 titers against VRC07.523, PGT121, and PGDM1400, for breastmilk viruses, contemporaneous adult viruses, and infant viruses. Two sets of breastmilk viruses are considered: the virus most sensitive to a given bnAb, among all viruses sequenced from each mother; and the virus most resistant to a given bnAb.

bioRxiv preprint doi: https://doi.org/10.1101/2025.11.04.686466; this version posted November 4, 2025. The copyright holder for this preprint (which was not certified by peer review) is the author/funder, who has granted bioRxiv a license to display the preprint in perpetuity. It is made available under aCC-BY 4.0 International license.

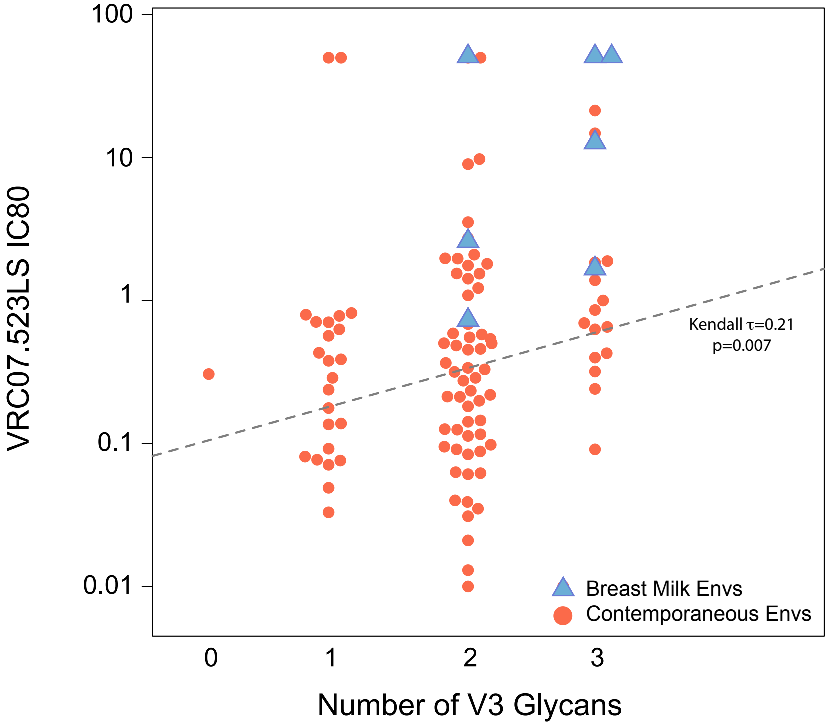

Fig. S3. Correlation between the number of glycosylation sites in V3 and VRC07.523 resistance. The 7 VRC07.523 most resistant BM envs are shown in light blue triangles while the 99 contemporaneous clade C viruses are shown in orange circles.

bioRxiv preprint doi: https://doi.org/10.1101/2025.11.04.686466; this version posted November 4, 2025. The copyright holder for this preprint (which was not certified by peer review) is the author/funder, who has granted bioRxiv a license to display the preprint in perpetuity. It is made available under aCC-BY 4.0 International license.

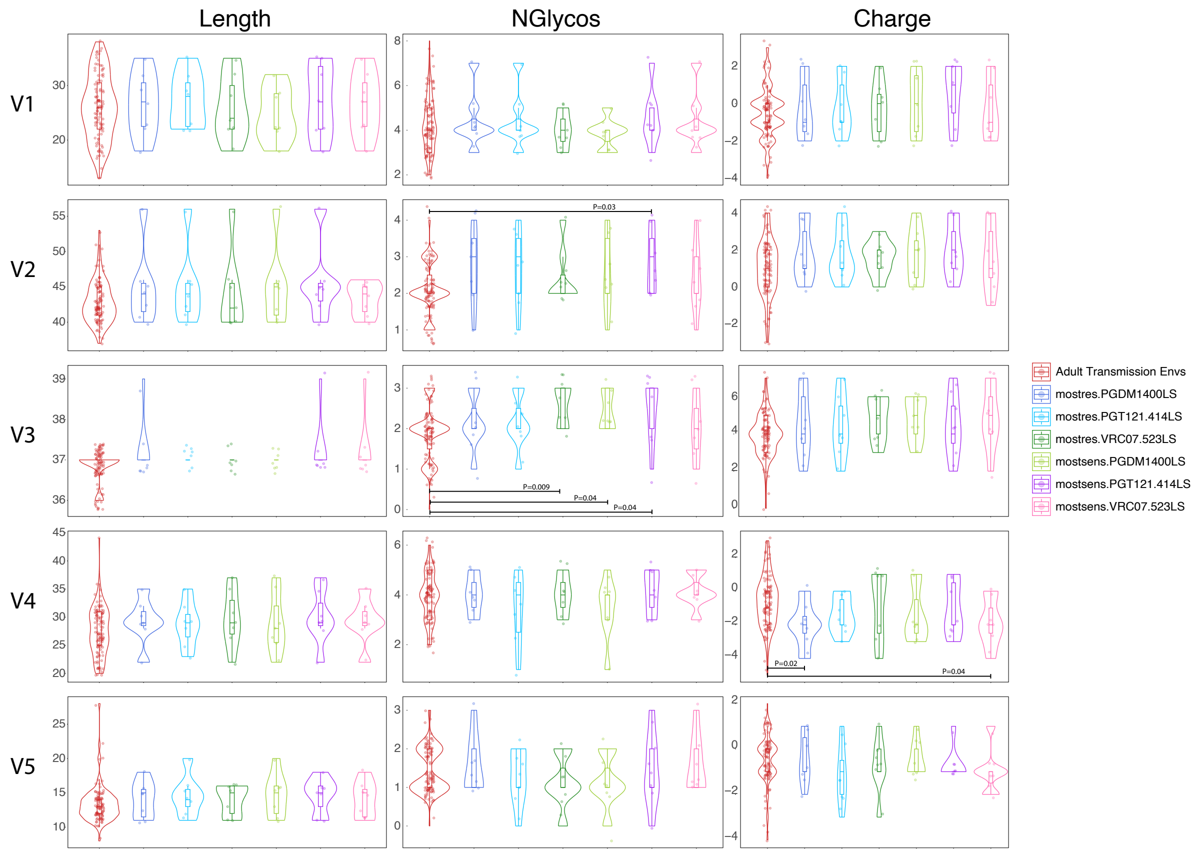

Fig S4. Comparisons of length, charge, and number of glycosylation sites at the five variable regions V1-V5. Each violin plot represents a different dataset. Statistically significant comparisons are marked with the corresponding p-value. All other comparisons were not statistically significant at a 0.05 significance threshold.
